# Plasma Homoarginine Concentrations According to Use of Hormonal Contraception

**DOI:** 10.1038/s41598-018-30708-y

**Published:** 2018-08-15

**Authors:** Thea Helm, Kristin Varsi, Christina Herland Fløtre, Agnethe Lund, Gard Frodahl Tveitevåg Svingen, Per Magne Ueland, Anne-Lise Bjørke-Monsen

**Affiliations:** 1 Faculty of Medicine, University of Bergen, Bergen, Norway; 2 Laboratory of Clinical Biochemistry, Haukeland University Hospital, Bergen, Norway; 3 Department of Obstetrics and Gynecology, Haukeland University Hospital, Bergen, Norway; 4 Department of Heart Disease, Haukeland University Hospital, Bergen, Norway; 5 Department of Clinical Science, Faculty of Medicine, University of Bergen, Bergen, Norway

## Abstract

Estrogen is a potent vasodilator through activation of endothelial nitric oxide synthase (eNOS). Arginine and its homologue homoarginine are substrates for NOS, while asymmetric dimethylarginine (ADMA) is a NOS inhibitor. Healthy, never-pregnant women aged 18 to 40 years (n = 158) were categorized according to use of hormonal contraception into non-users (n = 76), users of estrogen contraceptives (EC-users, n = 58) and users of progestins-only contraceptives (PC-users, n = 24). Plasma homoarginine, arginine, ADMA and SDMA concentrations were assayed using a LC-MS/MS method. Compared to non-users, EC users had higher plasma homoarginine (median (interquartile range) 1.63 (1.24, 2.04) vs. 2.39 (2.05, 2.85) µmol/L, p < 0.001), lower arginine (80.8 (72.4, 94.3) vs. 72.1 (62.9, 85.1) µmol/L, p = 0.008) and ADMA (0.52 (0.46, 0.59) vs. 0.48 (0.42, 0.54) µmol/L, p = 0.003) concentrations. The lowest median plasma homoarginine concentration (1.34 (0.92, 1.75) µmol) was seen in PC-users. No differences were seen in SDMA concentrations according to use of hormonal contraception. In healthy, never-pregnant women aged 18 to 40 years, use of estrogen containing contraception was associated with significantly higher plasma concentrations of homoarginine and lower plasma concentrations of arginine and ADMA as compared to non-users, while the lowest plasma homoarginine concentrations were seen in progestin-only users. Whether the observed changes in relation to use of hormonal contraception have an impact on cardiovascular status, should be evaluated in an intervention study.

## Introduction

Estrogen is a potent vasodilator, and conditions with high estrogen concentrations, as during the latter part of the follicular phase of the menstrual cycle, use of oral contraception (OC) and pregnancy, are all associated with increased endothelial dependent vasodilatation^1–3^. Estrogen mediates its effect on the vascular endothelium partly via activation of endothelial nitric oxide synthase (eNOS)^4^. The resulting increase in circulating nitric oxide (NO) is crucial for endothelial function, including arteriolar relaxation, an important determinant of blood pressure^5^. L-Arginine and its homolog L-homoarginine are competitive substrates of NOS^6^, whereas asymmetric dimethylarginine (ADMA), a guanindine (*N*^*G*^)-dimethylated derivate of arginine, is a NOS inhibitor^7^. ADMA and the stereoisomer symmetric dimethylarginine (SDMA) may also indirectly reduce NO synthesis by inhibiting cellular uptake of arginine^8^. Accordingly, the ratio between arginine and ADMA is regarded as a marker of NOS activity^7^. A favourable cardiovascular risk profile has been related to high circulating homoarginine^9^ and low ADMA concentrations^10^.

Higher arginine and lower ADMA concentrations are reported in women using OC compared to non-users^11^, while lower arginine and ADMA concentrations are reported in pregnant women^12^. A marked increase in homoarginine concentrations are observed in women using OC^13^ and and in pregnant women during the second and third trimester. In pregnancy, homoarginine concentrations are reported to be more strongly correlated to brachial artery flow-mediated dilatation than arginine^12^.

We investigated systemic amino acids involved in NO regulation in healthy, never-pregnant women aged 18 to 40 years. The purpose of the study was to evaluate plasma homoarginine, arginine, ADMA and SDMA concentrations in relation to use of hormonal contraception.

## Results

### Demographics

The population included never-pregnant women with a mean (range) age of 25.3 (18–40) years. The participants were healthy, well-educated, with a median (IQR) BMI of 21.8 (20.6, 23.7). The majority (124/158, 78%) had an omnivore diet, and a minority (21/158, 30%) were regular users of both multivitamins/minerals and omega 3 fatty acids/cod oil supplements.

Demographic data according to reported current use of contraceptives (non-users, n = 76, EC-users, n = 58 and PC-users, n = 24) are given in Table 1. Apart from a higher consumption of alcohol in women who used hormonal contraceptives compared to non-users, there were no significant differences in demographic data among the three groups (Table 1).

**Table 1:** Baseline characteristics of healthy, never-pregnant women according to use of contraceptives (n = 158).

	Non-users N = 76	Users of hormonal contraceptives		P value
		With estrogen and progestins N = 58	Progestin-only N = 24	
Age, y, mean (SD)	25.6 (5.6)	24.9 (4.1)	24.8 (4.0)	0.61*
BMI, kg/m^2^, median (IQR)	22.2 (20.8, 24.5)	21.0 (20.1, 21.8)	22.3 (20.6, 23.6)	0.13**
Higher education, n (%)				
<12 years	10 (13%)	4 (7%)	0 (0%)	0.09***
13–17 years	19 (25%)	24 (41%)	7 (29%)	
>17 years	47 (62%)	30 (52%)	17 (71%)	
Vegetarian diet, n (%)	17 (22%)	11 (19%)	6 (25%)	0.81***
Regular users of supplements, (≥3 days/week), n (%)				
Multivitamins/minerals	15 (20%)	14 (24%)	6 (25%)	0.80***
Omega 3 fatty acids/Cod oil	30 (40%)	30 (52%)	10 (42%)	0.35***
Iron	11 (15%)	9 (16%)	2 (8%)	0.68***
Regular users of tobacco, based on plasma cotinine >85 µmol/L, n (%)	8 (11%)	7 (12%)	2 (8%)	0.87***
Alcohol, number of units/week, median (IQR)	1.0 (0.3, 3.0)	2.0 (1.0, 4.0)	2.8 (1.0, 4.0)	0.006**

*Comparison by Anova test.

**Comparison by Kruskal Wallis test.

***Comparison by Pearson Chi-Square test.

### Plasma homoarginine, arginine, ADMA and SDMA concentrations according to use of hormonal contraceptives

**Figure 1:**
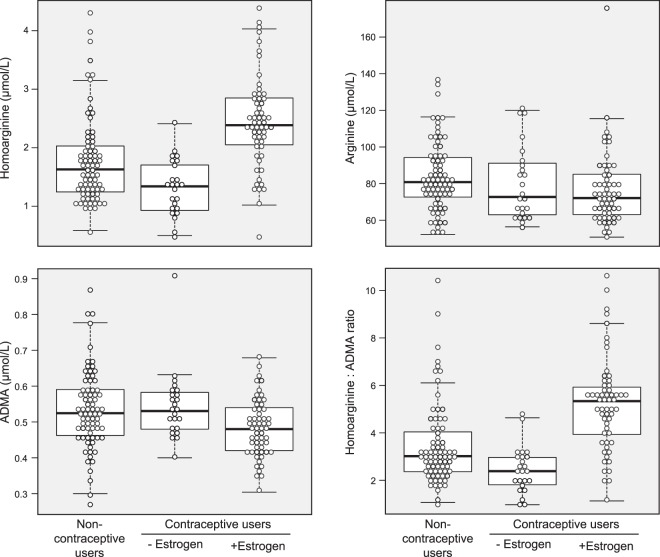
Plasma homoarginine, arginine and ADMA concentrations and hArg/ADMA ratio in relation to use of hormonal contraception.

There were significant differences in the plasma concentrations of homoarginine, arginine and ADMA, but not in SDMA, according to use of hormonal contraception (Table 2, Fig. 1). Plasma homoarginine concentrations were higher in EC-users (+47%, p < 0.001) and lower in PC-users (−18%, p = 0.005), as compared to non-users (Table 2). The majority of the EC-users (13/58, 78%) had homoarginine concentrations >2.0 µmol/L, while the majority of the non-users (56/76, 74%) and almost all PC-users (23/24, 96%) had concentrations <2.0 µmol/L.

**Table 2:** Plasma concentrations of homoarginine, arginine, ADMA, SDMA and Arg/ADMA and hArg/ADMA ratios according to use of contraceptives (n = 158).

Parameters	Non-users N = 76	Users of hormonal contraceptives		P value*
		With estrogen and progestins N = 58	Progestin-only N = 24	
Plasma homoarginine, µmol/L	1.63 (1.24, 2.04)	2.39 (2.05, 2.85)	1.34 (0.93, 1.73)	<0.001
Plasma arginine, µmol/L	80.8 (72.4, 94.3)	72.1 (62.9, 85.1)	72.7 (62.8, 91.8)	0.03
Plasma ADMA, µmol/L	0.52 (0.46, 0.59)	0.48 (0.42, 0.54)	0.53 (0.48, 0.58)	0.003
Plasma SDMA, µmol/L	0.54 (0.46, 0.61)	0.56 (0.49, 0.59)	0.52 (0.49, 0.60)	0.86
Arg/ADMA ratio	157.5 (134.8, 178.9)	156.4 (138.5, 178.3)	141.3 (123.8, 168.5)	0.24
hArg/ADMA ratio	3.02 (2.36, 4.09)	5.34 (3.86, 5.93)	2.39 (1.78, 2.99)	<0.001

*Median (IQR), comparison by Kruskal Wallis test.

EC-users had lower median arginine and ADMA concentrations compared to non-users (p = 0.008 and p = 0.003, for arginine and ADMA respectively), and lower ADMA concentrations compared to PC-users (p = 0.004).

EC-users had higher median homoarginine/ADMA (hArg/ADMA) ratio compared to both non-users and PC-users (p < 0.001), while the lowest ratio was seen in PC-users. No significant differences in median arginine/ADMA (Arg/ADMA) ratios were observed between the groups (Table 2).

Use of hormonal contraception was the strongest positive predictor of homoarginine and the hArg/ADMA ratio, and the strongest negative predictor of ADMA concentrations, in a multiple linear regression model which additionally included age, BMI, use of alcohol and tobacco (based on cotinine levels ≥85 nmol/L) (Table 3).

**Table 3:** Determinants of plasma amino acids concentrations in healthy never-pregnant women (n = 158) by multiple linear regression.

Variables included in the model	Homoarginine		Arginine		ADMA		SDMA		Arg/ADMA		hArg/ADMA	
	*Beta*	P	*Beta*	P	*Beta*	P	*Beta*	P	*Beta*	P	*Beta*	P
Use of contraception*	0.41	<0.001	−0.13	0.13	−0.18	0.03	−0.00	1.00	−0.02	0.82	0.42	<0.001
Age	0.06	0.47	0.05	0.55	−0.16	0.06	−0.05	0.60	0.16	0.08	0.07	0.35
BMI	0.16	0.05	−0.08	0.34	−0.07	0.42	−0.05	0.60	−0.03	0.78	0.14	0.07
Alcohol intake**	−0.15	0.07	−0.01	0.94	−0.01	0.88	0.08	0.36	−0.01	0.90	−0.14	0.09
Regular use of tobacco***	0.02	0.93	−0.03	0.72	−0.03	0.74	−0.05	0.59	−0.01	0.93	0.02	0.82

^*^Use of contraception: Non-user, user of progestin-only contraception, user of estrogen and progestins containing contraception.

^**^Number of alcohol units/week.

^***^Based on plasma cotinine concentrations, categorized into <85 nmol/L or ≥85 nmol/L.

## Discussion

In healthy, never-pregnant women aged 18 to 40 years, the use of estrogen containing contraception was associated with significantly higher plasma concentrations of homoarginine, higher hArg/ADMA ratio and lower concentrations of arginine and ADMA as compared to non-users. The lowest homoarginine concentrations were seen in progestin-only users. No difference in Arg/ADMA ratio was seen according to use of hormonal contraception.

Premenopausal women who do not use hormonal contraception, have the highest circulating estrogen concentrations in the latter part of the follicular phase, just prior to ovulation^14^. Oral contraceptive pills are classically prescribed as a constant supply of estrogen for 21 days, followed by 7 days of placebo^15^. The transdermal patch or vaginal ring is worn for 21 days when it delivers a continuous estrogen and progestin formulation and is then removed for 7 days^16^. As a result, women who use combination contraceptives have a constant high estrogen concentration for 21 of a 28 days cycle, and a lower estrogen concentration during the 7 days with placebo. The various forms and doses of progestins in progestin-only contraceptives differ in their inhibition of ovarian activity^17^. The estrogen levels differ accordingly, but are reported to be lower or remain comparable to normal early or mid-follicular phase levels^17–19^. In pregnant women, the estrogen concentrations increase continuously during pregnancy with the highest concentrations observed in the last trimester^20^.

Homoarginine is endogenously synthesized by L-arginine:glycine amidinotransferase (AGAT)^13^, and gene expression is shown to be modulated by estrogen in chick liver cells^21^. Conditions with higher estrogen concentrations are associated with higher circulating homoarginine concentrations, as confirmed in the present and previous studies on oral oestrogen contraceptive users^13^ and also in pregnant women^12^.

The majority (78%) of the EC-users in our population had high homoarginine concentrations (>2 µmol/L), but in one quarter of the sample the concentration was below 2 µmol/L. One quarter of the non-users had a high homoarginine concentration (>2 µmol/L). The observed variations could be explained by cyclic changes in estrogen concentrations in our population. Assuming this was a representative sample of premenopausal women, one would expect one quarter of the EC-users to be in their placebo-phase (7/28 days) with low estrogen concentrations and one quarter of the non-users to be in their late follicular phase with high estrogen concentrations.

The lowest homoarginine concentrations were seen in PC-users. This might be explained by lower estrogen levels in PC-users compared to non-users and EC-users; however, as various progestins have different effects on ovarian activity, this may also reflect specific progestin subtypes and doses. Progestins might also have an independent effect on endogen homoarginine production, but this is currently unknown.

Arginine concentrations are reported to be higher in in the follicular phase compared to the luteal phase^11^, although one study did not observe any changes in arginine concentrations during the menstrual cycle^12^. Lower ADMA concentrations have been reported in conditions associated with higher estrogen concentrations, as in pregnancy^22^, after ovarian hyperstimulation^23^ and after hormone therapy in postmenopausal women^24^, which are observations in agreement with the results in the present study. We observed no differences in SDMA according to hormonal contraceptive use.

In our population of fertile women, the hArg/ADMA ratio was the strongest predictor of hormonal contraception use, with the highest ratio seen in EC-users, followed by non-users and PC-users. Association of hArg/ADMA ratios with higher estrogen levels are also reported by Valtonen *et al*. Nonpregnant women had a hArg/ADMA ratio of 5, while the values increased from 6 to 10 during pregnancy^12^. The hArg/ADMA ratio is considered to be mainly determined by circulating concentrations of homoarginine, which shows a larger variation than ADMA that is more closely controlled^25^.

We observed no differences in Arg/ADMA ratio. The Arg/ADMA ratio was reported to be higher in OC-users compared to non-users in one study^11^, while no change was seen in postmenopausal women after hormone replacement^24^. Slightly higher Arg/ADMA ratios have been found in pregnant women in their second and third trimester, although this was not related to improved endothelial function^22^. The Arg/ADMA ratio may reflect the capacity of NOS catalysed NO formation from arginine; however, as the ratio is mainly determined by large fluctuations in circulating concentrations of arginine, the utility of this ratio has been questioned^25^.

Physiological states associated with high estrogen concentrations are associated with increased endothelial dependent vasodilatation^1–3^. In premenopausal women with variant angina, the frequency of ischemic episodes was lowest in the late part of the follicular phase^26^, associated with the highest estrogen concentrations in the menstrual cycle^20^. Use of estrogen containing contraceptives has also been shown to cause vasodilatation^2,3,27^. These estrogen effects are probably mediated by increased expression and activation the endothelial isoform of NO synthase (eNOS)^4^, which uses both arginine and homoarginine as substrates for NO production^6^.

A higher cardiovascular risk has been related to high ADMA^10^, high SDMA^28^ and low circulating homoarginine concentrations^9,25^. High circulating homoarginine concentrations have also been associated with cardiovascular risk factors like hypertension, obesity and insulin resistance^13^. A 10 year follow-up study of young Finnish adults reported no effect of lifetime exposure of higher homoarginine levels on cardiovascular disease risk^13^, so the role for homoarginine as a biomarker for cardiovascular status still remains uncertain in young adults^13^.

ADMA and homoarginine are considered to have opposite effects on NO production, with ADMA serving as an inhibitor of NOS and homoarginine as a substrate^29^. The vascular effects of ADMA and SDMA have however been questioned, as ADMA and presumably also SDMA, are considered to be weak inhibitors of eNOS^25^. Homoarginine has a low affinity for NOS^30^, but is considered an alternative substrate to arginine and has been shown to increase NO availability^31^. Homoarginine is however, also reported to reduce NO production by acting as an inhibitor rather than a substrate for the three NO synthase isoforms (eNOS, neuronal and inducible NOS)^32^. Homoarginine may compete with arginine at the substrate binding site^33^ and has been shown to impair cellular arginine transport^34^ by inhibiting the cationic amino acid transporter (CAT-1), thereby reducing intracellular arginine availability for NOS^35^, and possibly also for other enzymatic reactions. However, high concentrations of L-homoarginine (1 mmol/L) were used to inhibit arginine uptake^34^ and it is unlikely that such levels can be reached in humans. Therefore, the exact mechanisms for homoarginine inhibition of NO production are still not clear.

Arginine is an important amino acid in pregnancy and serves both as a building block for proteins and is additionally hydrolyzed by the enzyme arginase to ornithine and converted into the polyamines putrescine, spermine, and spermidine^36^,which are key regulators of placental angiogenesis, trophoblast growth, and embryogenesis^37^. Arginine is reported to be abundant in the amniotic fluid in early pregnancy^38^, and a predictor for birth weight, length, and head circumference^39^.

The study included 158 healthy never-pregnant premenopausal women, of whom approximately 50% used hormonal contraception, which was a sufficient population size to detect statistically significant differences in concentrations of arginine metabolites studied. As we did not have information about the menstrual phase, sex hormone concentrations, or endothelial dependent vasodilatation, we were unable to relate our data to estrogen concentrations and vascular effects more directly, which are all limitations of this study.

## Conclusion

In healthy, never-pregnant women aged 18 to 40 years, use of estrogen containing contraception was associated with significantly higher plasma concentrations of homoarginine and lower concentrations of arginine and ADMA compared to non-users. The lowest homoarginine concentrations were seen in progestin-only users.

Whether the observed changes in arginine and its metabolites in relation to use of hormonal contraception have an impact on cardiovascular health should be evaluated in an intervention study.

## Material and Methods

### Study population and design

Between June 2012 and March 2015, healthy, never-pregnant women aged 18 to 40 years were recruited among employees and students at Haukeland University Hospital and the University of Bergen, Norway.

Ethical approval of the protocol was granted by the Regional Committee for Medical Research Ethics West (2011/2447). All methods were performed in accordance with the relevant guidelines and regulations. Written informed consent was obtained from all women.

The dataset generated and analyzed for the current study are available from the corresponding author on reasonable request.

### Clinical data

The women completed a questionnaire concerning age, body weight, health status, years of completed education, diet, and the use of multiple micronutrient supplements (MMN), alcohol and tobacco. Regular use of supplements was defined as use more than three days per week and the definition of a regular tobacco user was based on a plasma cotinine concentration >85 nmol/L^40^.

### Use of hormone containing contraceptives

Use of hormonal contraception, including oral contraceptives, hormone implants and injections, was recorded. Progestins-only contraceptives contain different forms and doses of progestins, while combination contraceptives additionally contain ethinylestradiol (range 20–35 µg)^15^.

Women who did not use hormonal contraception were defined as non-users, women who used estrogen containing contraceptives were defined as the EC-users, whereas women who used progestin-only contraceptives were defined as the PC-users.

### Blood sampling and analysis

Non-fasting blood samples were obtained by antecubital venipuncture and collected into EDTA Vacutainer Tubes (Becton Dickinson), placed in ice water, and plasma was separated within 4 hours. The samples were stored at −80 °C until analysis. Plasma concentrations of arginine, asymmetric dimethylarginine (ADMA), symmetric dimethylarginine (SDMA) and homoarginine were assayed using a LC-MS/MS method^41^ by the laboratory of Bevital AS (www.bevital.no).

### Statistical analysis

Results are presented as mean and standard deviation (SD), compared by Student’s t-test or ANOVA, and median and interquartile range (IQR), compared by Mann-Whitney U test or Kruskal Wallis test. Chi-square test was used for categorical data. Multiple linear regression models were used to assess the association of plasma homoarginine, arginine and ADMA with age, BMI, use of hormonal contraception, alcohol and tobacco.

The R Foundation for Statistical Computing (version 3.3) was used for graphical illustrations of the relation between use of hormonal contraception and homoarginine, arginine and ADMA. The SPSS statistical program (version 24) was used for statistical analyses. Two-sided p-values < 0.05 were considered statistically significant.
